# 20‐HETE synthesis inhibition attenuates traumatic brain injury–induced mitochondrial dysfunction and neuronal apoptosis via the SIRT1/PGC‐1α pathway: A translational study

**DOI:** 10.1111/cpr.12964

**Published:** 2020-12-13

**Authors:** Wenxing Cui, Xun Wu, Yingwu Shi, Wei Guo, Jianing Luo, Haixiao Liu, Longlong Zheng, Yong Du, Ping Wang, Qiang Wang, Dayun Feng, Shunnan Ge, Yan Qu

**Affiliations:** ^1^ Department of Neurosurgery Tangdu Hospital The Fourth Military Medical University Xi'an China

**Keywords:** 20‐HETE, apoptosis, mitochondrial dysfunction, SIRT1, traumatic brain injury

## Abstract

**Objectives:**

20‐hydroxyeicosatetraenoic acid (20‐HETE) is a metabolite of arachidonic acid catalysed by cytochrome P450 enzymes and plays an important role in cell death and proliferation. We hypothesized that 20‐HETE synthesis inhibition may have protective effects in traumatic brain injury (TBI) and investigated possible underlying molecular mechanisms.

**Materials and methods:**

Neurologic deficits, and lesion volume, reactive oxygen species (ROS) levels and cell death as assessed using immunofluorescence staining, transmission electron microscopy and Western blotting were used to determine post‐TBI effects of HET0016, an inhibitor of 20‐HETE synthesis, and their underlying mechanisms.

**Results:**

The level of 20‐HETE was found to be increased significantly after TBI in mice. 20‐HETE synthesis inhibition reduced neuronal apoptosis, ROS production and damage to mitochondrial structures after TBI. Mechanistically, HET0016 decreased the Drp1 level and increased the expression of Mfn1 and Mfn2 after TBI, indicating a reversal of the abnormal post‐TBI mitochondrial dynamics. HET0016 also promoted the restoration of SIRT1 and PGC‐1α in vivo, and a SIRT1 activator (SRT1720) reversed the downregulation of SIRT1 and PGC‐1α and the abnormal mitochondrial dynamics induced by 20‐HETE in vitro. Furthermore, plasma 20‐HETE levels were found to be higher in TBI patients with unfavourable neurological outcomes and were correlated with the GOS score.

**Conclusions:**

The inhibition of 20‐HETE synthesis represents a novel strategy to mitigate TBI‐induced mitochondrial dysfunction and neuronal apoptosis by regulating the SIRT1/PGC‐1α pathway.

## Graphical abstract

The mechanism on 20‐HETE–induced alterations of mitochondrial dynamics in neurons. 20‐HETE is synthesized from AA by CYP4A, which is inhibited by HET0016. The alterations of mitochondrial dynamics induced by 20‐HETE activate oxidative stress and subsequently result in neuronal apoptosis mediated by inhibition of the Sirt1/PGC‐1α signalling pathway.

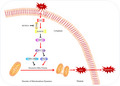

## 1 INTRODUCTION

Traumatic brain injury (TBI) is a leading cause of death and disability worldwide. Alleviation of secondary brain injury and promotion of neurological recovery after TBI are key to reducing the burden on affected families and society and to improving the quality of life of patients.^1^, ^2^ TBI involves two pathological processes: primary brain injury and secondary brain injury. Primary brain injury refers to brain contusion, subarachnoid haemorrhage and parenchymal haemorrhage, while secondary injury involves cerebral oedema and the damaging effects of excessive inflammatory responses, oxidative stress and abnormal mitochondrial activity.^3^, ^4^, ^5^ Secondary injury contributes significantly to progressive pathophysiological exacerbation and can affect neurological function; thus, strategies targeting the critical pathological factors that contribute to secondary brain injury are of great importance from the perspective of both basic science and clinical research.

Traumatic brain injury is known to induce the release of arachidonic acid (AA) from cell membranes.^6^ AA can be converted by cytochrome P450 (CYP) enzymes to hydroxyeicosatetraenoic acids (HETEs) and epoxyeicosatrienoic acids (EETs), and 20‐HETE is the major bioactive form.^7^, ^8^ Several studies have suggested that it has important physiological and pathological functions in the regulation of cell proliferation, inflammatory responses, vasoconstriction and angiogenesis.^9^, ^10^, ^11^, ^12^ HET0016 is a highly selective inhibitor of 20‐HETE synthesis and significantly reduces 20‐HETE levels.^13^ 20‐HETE–targeted therapies attenuate neuronal death and have been shown to improve neurological outcomes after cerebral ischaemia/reperfusion injury and intracerebral haemorrhage.^14^, ^15^ Studies have demonstrated that the level of 20‐HETE is increased in the plasma of patients with ischaemic or haemorrhagic stroke.^16^, ^17^ Moreover, elevated plasma levels of 20‐HETE are associated with unfavourable outcomes and may be a predictor of neurological deterioration in stroke patients.^17^, ^18^, ^19^ Thus, 20‐HETE may be a promising therapeutic target, and HET0016 treatment may protect neurons from TBI.

Mitochondrial injury or dysfunction can cause excessive oxidative stress after TBI and leads to continuous brain damage.^20^ Mitochondrial dynamics are mainly controlled by the division and fusion of mitochondria. Under pathological conditions, the energy homeostasis is disrupted, and excessive mitochondrial fission leads to oxidative stress and cell death.^21^ Peroxisome proliferator–activated receptor‐γ coactivator‐1α (PGC‐1α) is a master regulator of mitochondrial biogenesis, metabolism and antioxidant defences, and is known to contribute to neuronal survival.^22^ Sirtuin 1 (SIRT1) is the upstream regulator of PGC‐1α and its transcriptional activity,^23^ and is vital for the maintenance of energy homeostasis. Therefore, the SIRT1/PGC‐1α axis could be an important target in TBI. Furthermore, 20‐HETE has been shown to induce apoptosis via mitochondria‐dependent pathways^24^; however, the specific mechanism by which 20‐HETE may cause mitochondrial dysfunction has not been investigated.

In this study, we sought to determine whether 20‐HETE is involved in pathophysiological processes induced in a mouse model of TBI. We also investigated whether mitochondrial injury or dysfunction contributes to post–TBI 20‐HETE–induced apoptosis in neurons, focusing mechanistically on the SIRT1/PGC‐1α pathway. Finally, plasma 20‐HETE levels and their association with TBI outcomes were assessed in patients with TBI.

## 2 METHODS

### 2.1 Animals and ethical considerations

Male wild‐type (WT) C57BL/6 mice (20‐25 g, 8‐12 weeks old) were purchased from the Animal Center of the Fourth Military Medical University. Animals were housed in a specific pathogen‐free environment animal room with a 12‐h light/dark cycle, a constant temperature of 23°C and humidity of 60%. The animals had ad libitum access to water and food from at least 1 week before the experiments. All experimental procedures were approved by the Ethics Committee of the Fourth Military Medical University. Animal experiments were performed in accordance with the guidelines of the National Institutes of Health Guide for the Care and Use of Laboratory Animals.

### 2.2 TBI surgery, drug administration and experimental design

The TBI model was established using a controlled cortical impact (CCI) device (68099 precision strike; RWD). Briefly, the mice were anaesthetized using 2% pentobarbital sodium, and the head was fixed on a stereotactic device. Craniotomy was performed with over the right parietal bone window (1.5 mm towards the midline and 1.5 mm behind the bregma) using a 2‐mm‐diameter dental drill. A flat metal tip was used to strike the cortex at a speed of 3 m/s and depth of 1.8 mm; the contact time was 2 s. Cyanoacrylate tissue glue was used to close the scalp. The sham control group underwent craniotomy but did not receive CCI injury. Mice were blindly randomized into different groups: (a) sham group, (b) TBI group, (c) TBI + vehicle group and (d) TBI + HET0016 group. HET0016 (HY‐124527, MCE) dissolved in 0.9% saline was used. Mice were randomly received HET0016 (1, 1.5 and 2 mg/kg) or vehicle at 2 hours after TBI.

### 2.3 ELISA

Mice were sacrificed 6, 12, 24, 48 or 72 hours after TBI, and brain samples were collected from the injured area. Levels of 20‐HETE in the mouse brain samples were determined using an ELISA Kit (ab175817, Abcam). Whole blood from patients with TBI was collected in EDTA‐treated collection tubes and centrifuged at 1,500 g for 15 min to obtain the plasma. Levels of 20‐HETE in the plasma samples were determined using a suitable ELISA Kit (JL18922, Jiang Lai). Levels of SIRT1 in the plasma samples were analysed by the ELISA Kit (ELH‐SIRT1, RayBio). All ELISA procedures were carried out according to the manufacturer's instructions.

### 2.4 Immunofluorescence and terminal deoxynucleotidyl transferase–mediated dUTP nick end labelling (TUNEL) staining

Coronal sections of the brain were stained by standard immunohistochemistry procedures as described previously.^25^ Briefly, the brain sections were incubated with PBS containing 0.1% Triton X‐100 and 5% goat serum (Gibco) for 30 minutes. Then, the sections were incubated overnight at 4°C with the following antibodies as appropriate: anti‐CYP4A (1:200; Abcam, ab3573), anti‐NeuN (1:200; Millipore, MAB377), anti‐GFAP (1:200; Invitrogen) and anti‐Iba1 (1:200; Invitrogen). Nuclei were labelled with DAPI (1:1000, Invitrogen). All sections were imaged using an A1 Si confocal microscope (Nikon) and analysed using ImageJ.

TUNEL staining was used to detect apoptosis and was performed according to the manufacturer's protocols (Roche). Briefly, the sections were treated with 0.3% hydrogen peroxide for 30 minutes and then incubated with proteinase K for 45 minutes. Thereafter, the sections were immersed in TUNEL reaction solution in the dark for 60 minutes. Finally, the sections were labelled using DAPI (1:1000; Invitrogen), and the ratio of TUNEL‐positive to DAPI‐stained cells was calculated to assess the degree of apoptosis.

### 2.5 Measurement of cerebral water content and quantification of lesion volume

The wet‐dry weight ratio method was used to measure the cerebral water content as described previously.^26^ The ratio was calculated as a percentage using the following equation: brain water content (%) = (wet weight − dry weight)/wet weight × 100%.

Lesion volumes were quantified as described previously.^27^ Mice were sacrificed 24 hours after TBI, and brain sections were prepared. Thereafter, Nissl bodies were stained using cresyl violet, and ImageJ was used to quantify the lesion volume in each brain section.

### 2.6 Functional neurological testing

Neurological function was measured using the Modified Neurologic Severity Score (mNSS) and the corner turn and wire‐hanging tests on days 1, 3 and 7 after TBI.^15^ The mNSS considers motor, sensory, reflex and balance‐related function; the score was graded from 0 (normal) to 18 (maximal deficiency). The tests were conducted by two blinded observers. For the corner turn test, mice were allowed to proceed into a 30° corner. Each mouse was tested 10 times, and the percentage of left turns was calculated. For the wire‐hanging test, a metallic wire (1 mm × 55 cm) was stretched horizontally 50 cm above the ground. The hindlimbs of the mice were covered with adhesive tape to prevent them from being able to use all four paws, and a pillow was placed beneath the hindlimbs. The mice were then placed on the wire, and the latency to fall was recorded. Latency‐to‐fall values less than 10 s were excluded from the calculations.

### 2.7 Determination of reactive oxygen species (ROS) levels

Dihydroethidium (DHE) staining was used to evaluate intracellular ROS levels in post‐TBI brains. Briefly, each brain was cut into 15‐μm‐thick sections, which were then stained using DHE (Yesen, 50102ES02) for 30 minutes and imaged using a laser scanning confocal microscope (A1 Si, Nikon). Assays for determining malondialdehyde (MDA) levels (BC0025, Solarbio) and manganese superoxide dismutase (MnSOD) activity (JL20470, Jiang Lai) were performed using commercial assay kits in strict accordance with the manufacturer's protocols.

### 2.8 Transmission electron microscopy

Mice were anaesthetized 24 hours after TBI, and perfused brains were placed in ice‐cold Hanks' Balanced Salt Solution. Each brain was cut at the coronal position into 1‐mm‐thick sections. Cortical samples (1 × 2 mm^2^) were selected and fixed in 4% glutaraldehyde at 4°C overnight. After post‐fixation in 1% osmium tetroxide for 1 hour, the sections were dehydrated using a graded ethanol series and embedded in resin. An ultramicrotome was used to trim and section the embedded sample blocks, and the sections were then placed on 200‐slot grids coated with polyvinyl alcohol ester and imaged using a JEM‐1400 electron microscope (JEOL) with an electric coupling camera (Olympus).

### 2.9 Primary neuron culture cell treatments

Primary neurons were isolated from the foetal brain of WT C57BL/6 mice as described previously.^28^ Briefly, brain tissues were isolated from E16 mouse brains and digested in 0.25% trypsin/EDTA solution. Mixed neural cells were maintained in DMEM containing 10% foetal bovine serum, 1% penicillin and 1% L‐glutamate at 37°C in humidified 5% CO2 and 95% air for 4 hours. Neurobasal medium supplemented with B27, 1% penicillin and 1% streptomycin was used to replace the DMEM. Cytosine arabinoside was added to inhibit glial cell growth on the third and seventh days. The neurons were used for the experiments on day 14. Cells were exposed to 20‐HETE solution with the concentration of 10 μM for 24 hours, except for 20‐HETE + SRT1720 group, which were pretreated with SRT1720 (5 μM) for 2 hours.^29^

### 2.10 Western blotting

Western blotting was performed as described previously.^25^ Brain tissue was collected from the injured area and homogenized in ice‐cold lysis buffer containing phosphatase and protease inhibitors. The extracted proteins were separated by SDS‐PAGE and transferred onto PVDF membranes (Millipore). After blocking with 5% nonfat milk, the membranes were incubated with the following primary antibodies as appropriate: anti‐SIRT1 (1:1000; ab189494, Abcam); anti‐PGC‐1α (1:1000; 66369‐1‐Ig, ProteinTech); anti‐DRP1 (1:1000; D6C7, Cell Signaling); anti‐Bcl2 (1:1000; gtx100064, Gene Tex); anti‐Bax (1:1000; 50599‐2‐Ig, ProteinTech); anti‐cleaved caspase‐3 (1:1000; ab49822, Abcam); anti‐Nrf2 (1:1000; 16396‐1‐AP, ProteinTech); anti‐Mfn1 (1:1000; A9880, ABclonal); anti‐Mfn2 (1:1000; D2D10, Cell Signaling); anti‐cytochrome c (1:1000; wh118104, Wanleibio); anti‐SIRT2 (1;1000; 19655‐1‐AP, ProteinTech); anti‐SIRT3 (1:1000; 10099‐1‐AP, ProteinTech); anti‐SIRT4 (1:1000; 66543‐1‐Ig, ProteinTech); anti‐SIRT5 (1:1000; 15122‐2‐AP, ProteinTech); anti‐SIRT6 (1:1000; 67510‐1‐Ig, ProteinTech); anti‐SIRT7 (1:1000; 5360, Cell Signaling); and anti‐β‐actin (1:3000; wh096194, Wanleibio). The membranes were then incubated with the corresponding horseradish peroxidase–conjugated anti‐mouse or anti‐rabbit secondary antibodies (1:5000; AS003, AS014, ABclonal). Images were captured using a Bio‐Rad Imaging System (Bio‐Rad), and the protein bands were analysed using ImageJ.

### 2.11 Measurement of ATP levels and mitochondrial electron transport chain (ETC) complex activities

Mitochondria were isolated from freshly acquired brain samples using a Mitochondria Isolation Kit (C3606, Beyotime). ATP content (BC0300, Solarbio) and mitochondrial ETC (I‐IV) complex (BC0515, BC3230, BC3240, BC0945; Solarbio) activities were assayed using the appropriate commercially available kits according to the manufacturer's instructions.

### 2.12 Analysis of mitochondrial morphology and function

Mitochondrial morphology and function were analysed as described previously.^30^ Briefly, primary neurons were seeded on a confocal petri dish and subjected to the appropriated treatments. Thereafter, the cells were incubated with 10 nM MitoTracker Green (M7514, Life Technologies) in serum‐free cell culture medium for 30 minutes, and mitochondrial morphology was analysed using a fluorescent microscope (A1 Si, Nikon). Mitochondrial function analysis included measuring mitochondrial membrane potential (MMP) and mitochondrial ROS production. Cells were incubated with 10 nM JC‐1 (C2006, Beyotime) and 5 nM MitoSOX (M36008, Invitrogen) for 30 minutes. A fluorescent microscope was used to capture the images, and relative fluorescence levels were quantified using ImageJ.

### 2.13 Patients

This study was approved by the Ethics Committee of Tangdu Hospital, Fourth Military Medical University, and was conducted from September 2019 to April 2020. Emergency room patients aged 18‐80 years with mild, moderate or severe TBI were enrolled in the study. The exclusion criteria were any previous disabling neurological diseases or severe systemic diseases (uraemia, cirrhosis or malignant cancer). The primary outcome was 6‐month neurologic function, which was evaluated using the Glasgow Outcome Scale (GOS) score. GOS scores of 1‐3 were regarded as unfavourable functional outcomes, and GOS scores of 4‐5 were regarded as favourable functional outcomes as described previously.^31^

### 2.14 Statistical analysis

All statistical analyses were performed using IBM SPSS Statistics 20.0 (IBM). Continuous variables are presented as the median (interquartile range) if the distributions were skewed or as the mean ± standard deviation (SD) if normally distributed. Categorical data are presented as the frequency (percentage). A multivariate logistic regression model was used to identify independent risk factors for unfavourable 6‐month outcome. Spearman's rank correlation was used for univariate correlation analysis. Two independent groups were compared using the unpaired two‐tailed Student's t test, and one‐way analysis of variance was used to compare multiple groups. *P* < .05 was considered statistically significant.

## 3 RESULTS

### 3.1 Non‐targeted metabolic profiling indicates that the level of 20‐HETE is significantly increased after TBI in mice

**FIGURE 1:**
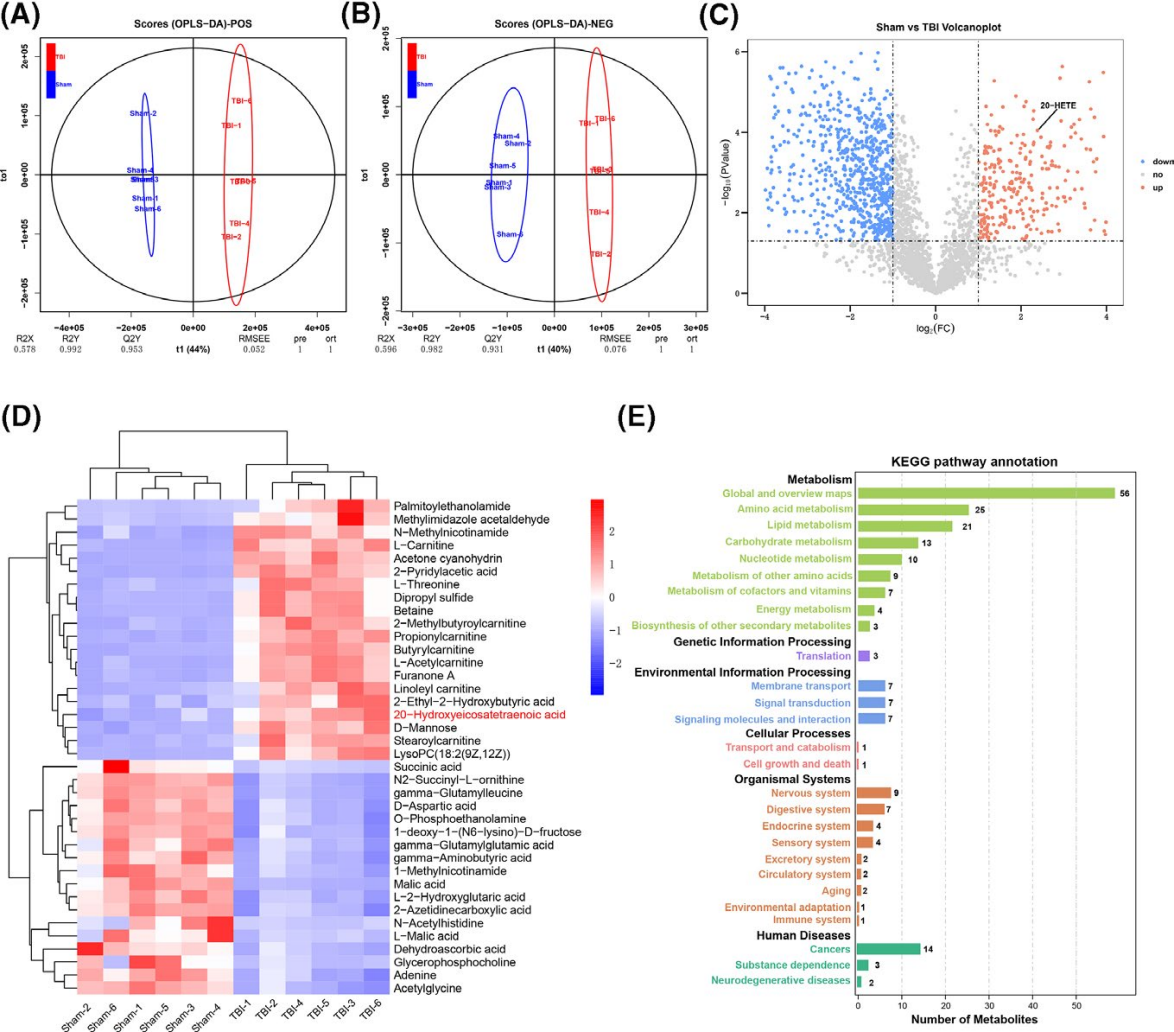
Non‐targeted metabolic profiling of damaged brain tissue after TBI. A, B, The clustering of orthogonal partial least‐squares discriminant analysis (OPLS‐DA). C, Volcano plot of up‐ and downregulated metabolites in sham and TBI groups. D, Heat map of the top 20 up‐ or downregulated metabolites in the two groups. E, Twenty‐seven KEGG pathways were significantly different between the two groups. n = 6 for each group

Non‐targeted metabolome analysis was used for differential metabolic profiling of TBI‐affected and normal brain tissue to identify potentially impactful molecules. Perilesional cortex samples from the TBI groups and normal cortex samples from the sham groups were differentiated using orthogonal partial least‐squares discriminant analysis (OPLS‐DA) (Figure 1A,B), indicating a differential metabolic profile. The volcano plot of the two groups shows all up‐ and downregulated metabolites (Figure 1C). The heat map shows the top 20 up‐ or downregulated metabolites (Figure 1D) and indicates a significant increase in 20‐HETE levels after TBI. Twenty‐seven Kyoto Encyclopedia of Genes and Genomes (KEGG) pathways were significantly different between the two groups (Figure 1E). The top three KEGG pathways were the global and overview maps, amino acid metabolism and lipid metabolism, indicating that lipid metabolism plays an important role in TBI. These results suggest that lipid metabolism is involved in the pathological process of TBI and that 20‐HETE may be a potential pathogenic factor.

### 3.2 Upregulation of 20‐HETE in mouse brain after TBI

**FIGURE 2:**
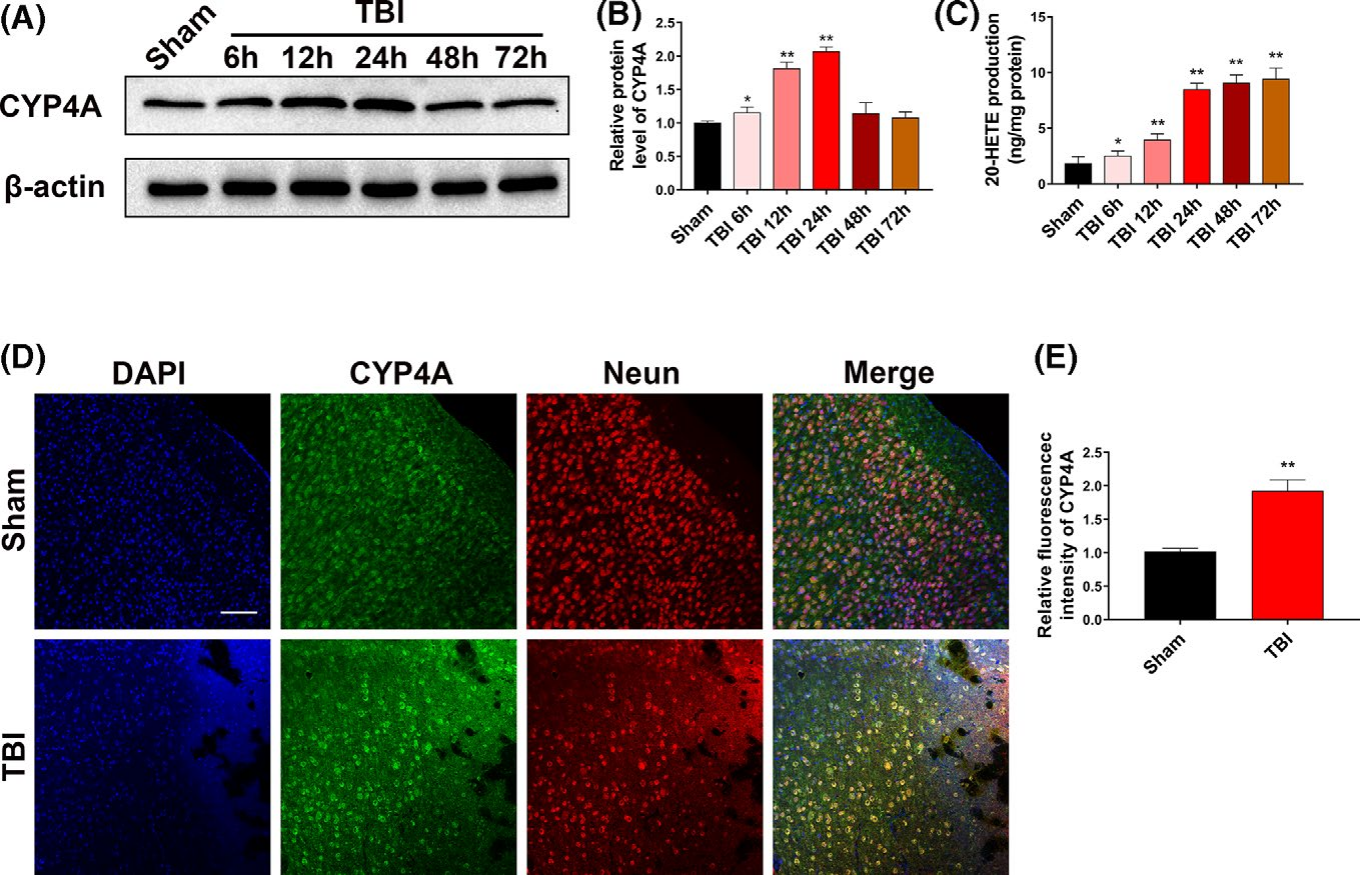
Induction of CYP4A expression and increase in 20‐HETE after TBI. A, B, Western blots and analysis of CYP4A levels in the perilesional cortex after TBI. C, Concentration of free 20‐HETE in perilesional cortex after TBI measured using ELISA. D, Distribution of NeuN^+^/CYP4A^+^ cells in the perilesional cortex 24 h after TBI. Green, CYP4A; red, NeuN. Scale bars: 100 μm. E, Quantitative analysis of fluorescence intensity of CYP4A (fold over Sham). n = 6 for each group. *P < .05 and **P < .01 vs sham group. Values are presented as the mean ± SD

To verify the changes in 20‐HETE levels after TBI, Western blotting was used to evaluate the possible changes in the levels of CYP4A, the 20‐HETE synthetase,^32^ in the perilesional cortex. CYP4A level was increased at 6 hours and reached a peak at 24 hours (Figure 2A,B). 20‐HETE levels in the perilesional cortex were assayed using ELISA. Compared with that in the sham group (1.82 ± 0.63 ng/mL), 20‐HETE levels were significantly upregulated at 6 hours (2.48 ± 0.46 ng/mL), 12 hours (3.95 ± 0.55 ng/mL), 24 hours (8.47 ± 0.58 ng/mL), 48 hours (9.04 ± 0.74 ng/mL) and 72 hours (9.39 ± 0.99 ng/mL) after TBI(Figure 2C). The 20‐HETE levels at 48 and 72 hours were not increased significantly compared with that at 24 hours. Thus, after 24 hours TBI was selected as the time point for subsequent experiments. Double immunofluorescence staining was performed to clarify the changes in the expression of CYP4A after TBI. In the intact brain, CYP4A was present primarily in neurons (Figure 2D). After TBI, CYP4A expression increased—staining was detected mainly in neurons, but also in astrocytes and microglia to a limited extent (Figure 2D,E; Figure S1A,B). These results indicate that 20‐HETE levels were significantly elevated in the perilesional cortex after TBI in mice and that the increased production of 20‐HETE was mainly derived from neurons.

### 3.3 HET0016 treatment mitigates brain injury, brain oedema and neurological functional deficits after TBI

**FIGURE 3:**
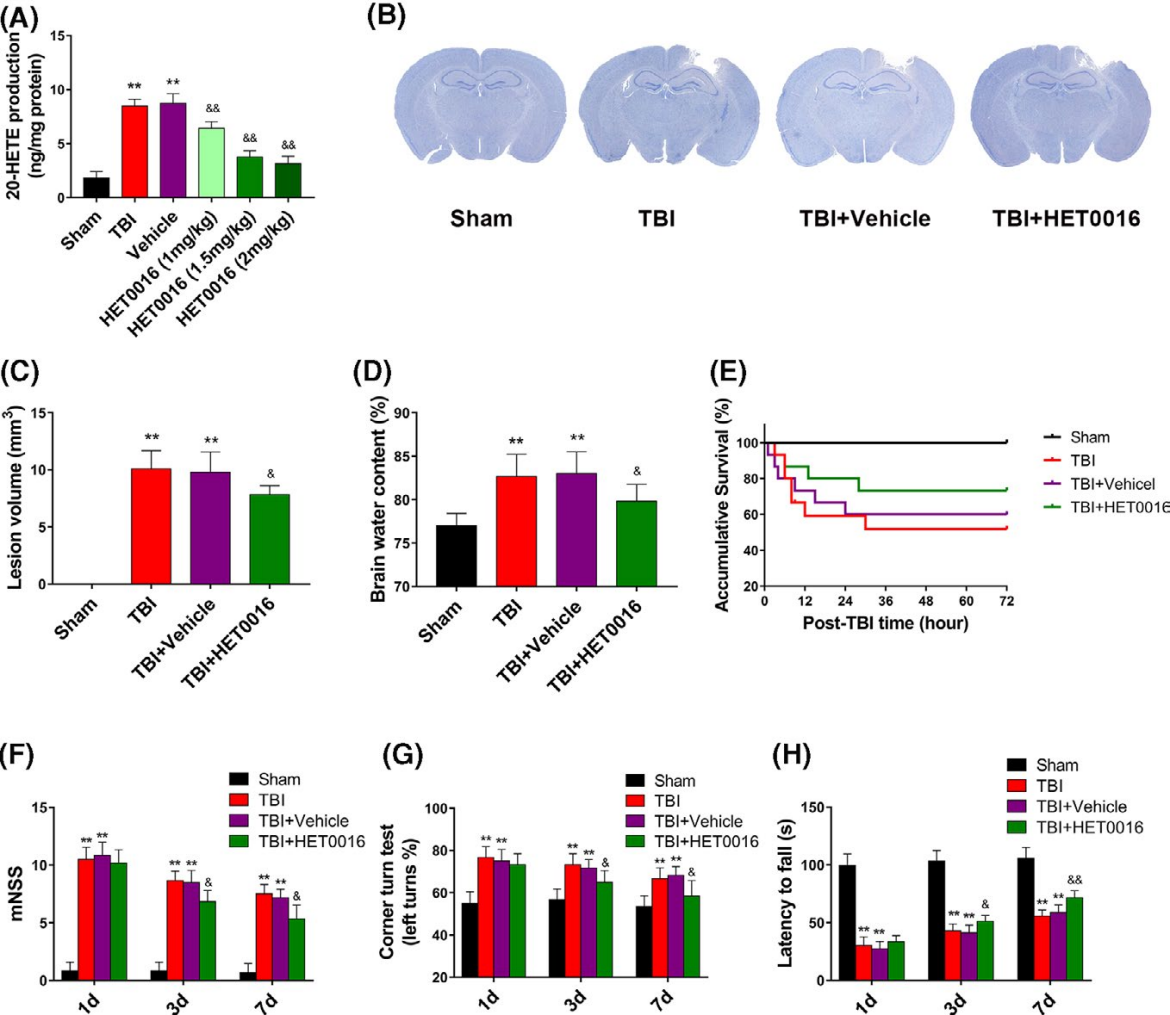
HET0016 reduces lesion volume and improves neurological outcome after TBI. A, The concentration of free 20‐HETE in the perilesional cortex after treatment with various doses of HET0016 (1, 1.5 and 2 mg/kg) 24 h after TBI. n = 6 for each group. B, Nissl staining of brain sections after TBI. n = 6 for each group. C, Intraperitoneal injection of HET0016 (1.5 mg/kg) reduced lesion volume. n = 6 for each group. D, HET0016 reduced brain oedema 24 h after TBI. n = 6 for each group. E, Kaplan‐Meier survival curves indicating lack of significant differences between the vehicle and HET0016 treatment groups. F, HET0016 reduced the neurological deficit score on day 3 and day 7 after TBI. n = 9 for each group. G, HET0016 improved the corner turn test performance of mice on day 3 and day 7 after TBI. n = 12 for each group. H, HET0016 increased latency to fall in the wire‐hanging test on day 3 and day 7 after TBI. n = 12 for each group. **P < .01 vs sham group, ^&^ *P* < .05 and ^&&^ *P* < .01 vs TBI + vehicle group. Values are presented as the mean ± SD

To validate the protective effects of 20‐HETE synthesis inhibition on brain injury, brain oedema and neurological functional deficits, mice were treated with HET0016, an inhibitor of 20‐HETE synthesis, after TBI. The optimal injection dose of HET0016 after TBI was determined, and 20‐HETE production was found to be minimized by a dose of 1.5 mg/kg (Figure 3A). Thus, a dose of 1.5 mg/kg was selected as the optimal administration dose for subsequent experiments. HET0016 treatment significantly reduced lesion volume and alleviated brain oedema at day 1 after TBI compared with those in the TBI and TBI + vehicle groups (Figure 3B‐D). However, HET0016 treatment did not significantly improve the survival of mice after TBI (Figure 3E). The neurological functions of mice at day 1, day 3 and day 7 after TBI were also evaluated. Mice treated with HET0016 had lower neurological functional deficit scores at day 3 and day 7 compared with those in the TBI and TBI + vehicle groups (Figure 3F). HET0016 treatment also improved performance in the corner turn test (Figure 3G) and increased the falling latency in the wire‐hanging test at days 3 and 7 after TBI (Figure 3H). These results suggest that HET0016 treatment has neuroprotective effects against TBI in mice.

### 3.4 HET0016 treatment alleviates oxidative stress and neural apoptosis after TBI

**FIGURE 4:**
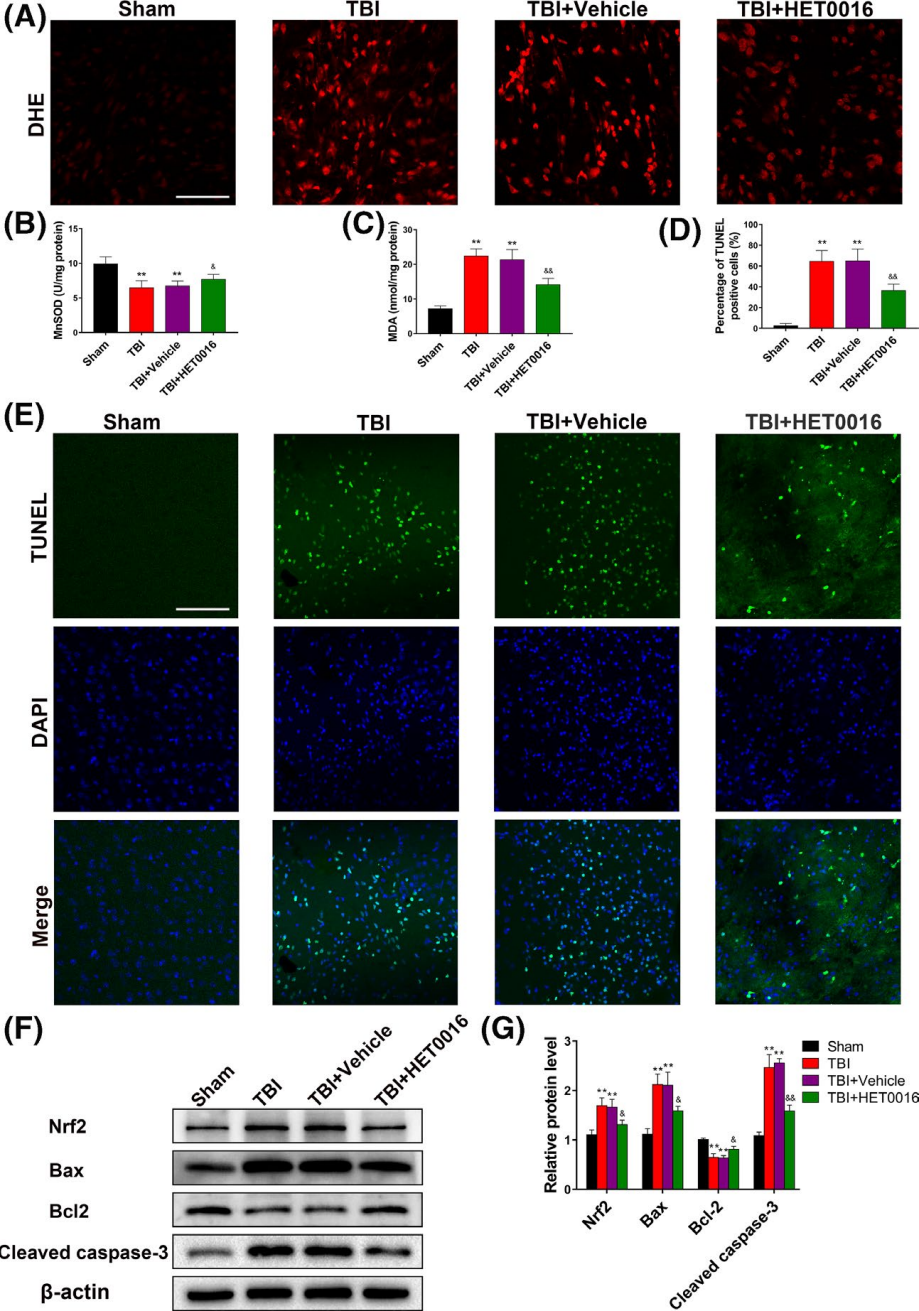
HET0016 alleviates oxidative stress and neural apoptosis after TBI. A, Representative images of DHE staining of the perilesional cortex 24 h after TBI, indicating the ROS levels. Scale bar: 100 μm. B, C, The effects of HET0016 on mitochondrial MnSOD activity and MDA levels. D, E, Representative images and statistical analysis of TUNEL staining of the perilesional cortex 24 h after TBI. Scale bar: 100 μm. F, G Representative Western blots and statistical analysis of the levels of Nrf2, Bax, Bcl‐2 and cleaved caspase‐3. n = 6 for each group. **P < .01 vs sham group, ^&^ *P* < .05 and ^&&^ *P* < .01 vs TBI + vehicle group. Values are presented as the mean ± SD

DHE fluorescence density in the TBI + HET0016 group was significantly lower than those in the TBI and TBI + vehicle groups (Figure 4A). Additionally, suppression of MnSOD activity and increase in MDA levels in the perilesional cortex after TBI were dramatically alleviated by HET0016 treatment (Figure 4B‐C). TUNEL staining indicated that HET0016 treatment dramatically reduced neural apoptosis after TBI (Figure 4D,E). Western blot results indicated a pronounced increase in the levels of Nrf2, Bax and cleaved caspase‐3 and a decrease in the level of Bcl2 after TBI, which were reversed by HET0016 treatment (Figure 4F,G). These results demonstrate that HET0016 treatment reduces oxidative stress and neural apoptosis after TBI.

### 3.5 HET0016 treatment protects the ultrastructure of neurons after TBI

**FIGURE 5:**
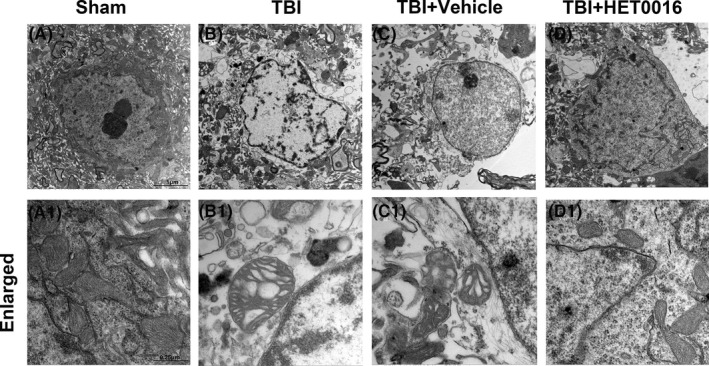
Effects of HET0016 on the ultrastructure of neurons in the perilesional cortex 24 h after TBI, as detected using electron microscopy. A–D, Representative ultrastructure of neurons in each group. A1‐D1, Magnified images of the ultrastructure of neurons shown in panels A‐D. Scale bar: 1 μm (A–D) and 0.25 μm (A1‐D1). n = 6 for each group

Transmission electron microscopy was used to observe the ultrastructural changes in neurons after TBI. In the sham group, the neurons had a normal shape, and the mitochondria were large and mostly oval, with a highly folded inner membrane that protruded inward to form the crista and a homogenous outer membrane that completely covered the organelle (Figure 5A,A1). In the TBI and TBI + vehicle groups, neurons were characterized by mitochondrial swelling and the disruption and disappearance of mitochondrial cristae and mitochondrial membrane integrity (Figure 5B‐C,B1‐C1). These pathological changes in the mitochondria were partially reversed by HET0016 treatment after TBI (Figure 5D,D1). These results indicate that 20‐HETE synthesis inhibition may protect neuronal mitochondrial structure from the damage induced after TBI.

### 3.6 HET0016 treatment reverses mitochondrial dysfunction via the SIRT1/PGC‐1α signalling pathway after TBI in vivo

**FIGURE 6:**
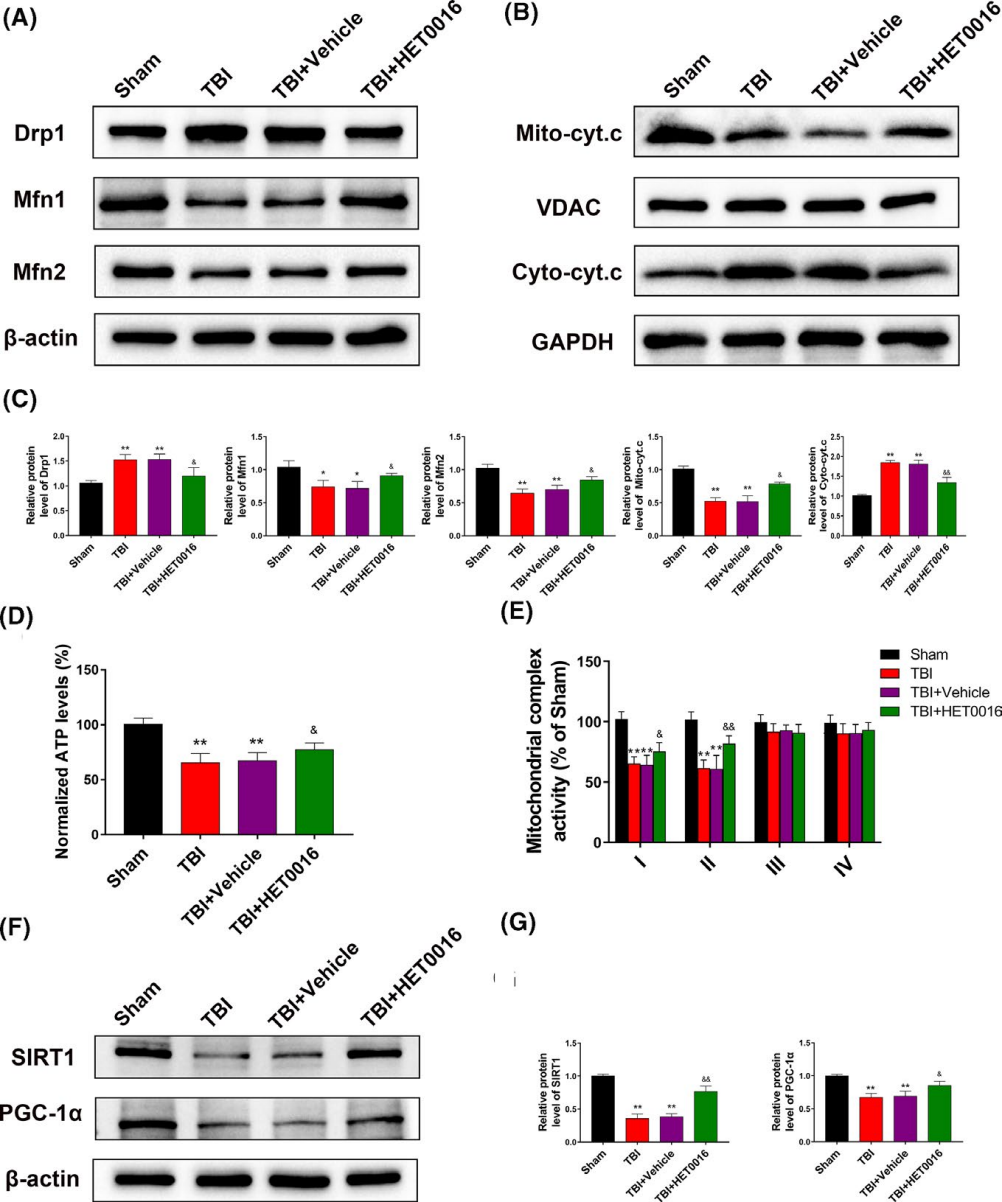
HET0016 reverses mitochondrial dysfunction via the SIRT1/PGC‐1α signalling pathway after TBI in vivo. A, C, Western blots and statistical analysis of the levels of Drp1, Mfn1 and Mfn2 in each group. B, C, Western blots and statistical analysis of the levels of mitochondrial and cytoplasmic cytochrome c in each group. D, Normalized ATP levels. E, Mitochondrial complex I, II, III and IV activities (percentage of sham). F, G, Western blots and statistical analysis of the levels of SIRT1 and PGC‐1α in each group. n = 6 for each group. *P < .05 and **P < .01 vs sham group, ^&^ *P* < .05 and ^&&^ *P* < .01 vs TBI + vehicle group. Values are presented as the mean ± SD

To better understand the potential mechanisms by which HET0016 restores mitochondrial function, we analysed the levels of proteins related to mitochondrial dynamics. The results showed that HET0016 treatment partially reversed the increase in the level of Drp1 and the decrease in the levels of Mfn1 and Mfn2 after TBI (Figure 6A,C). HET0016 treatment also inhibited the increase in the levels of cytosolic cytochrome c released from the mitochondria (Figure 6B,C), and ATP levels and mitochondrial complex I and complex II activities were increased in TBI mice treated with HET0016 (Figure 6D,E). Considering the pivotal roles of SIRT1 and PGC‐1α in mitochondrial homeostasis, we measured their levels and found them to be significantly reduced after TBI, which was partially reversed by HET0016 treatment (Figure 6F,G). These data suggest that HET0016 may regulate mitochondrial dynamics and function via the SIRT1/PGC‐1α signalling pathway.

### 3.7 20‐HETE disrupts mitochondrial morphology and function via regulation of the SIRT1/PGC‐1α pathway in vitro

**FIGURE 7:**
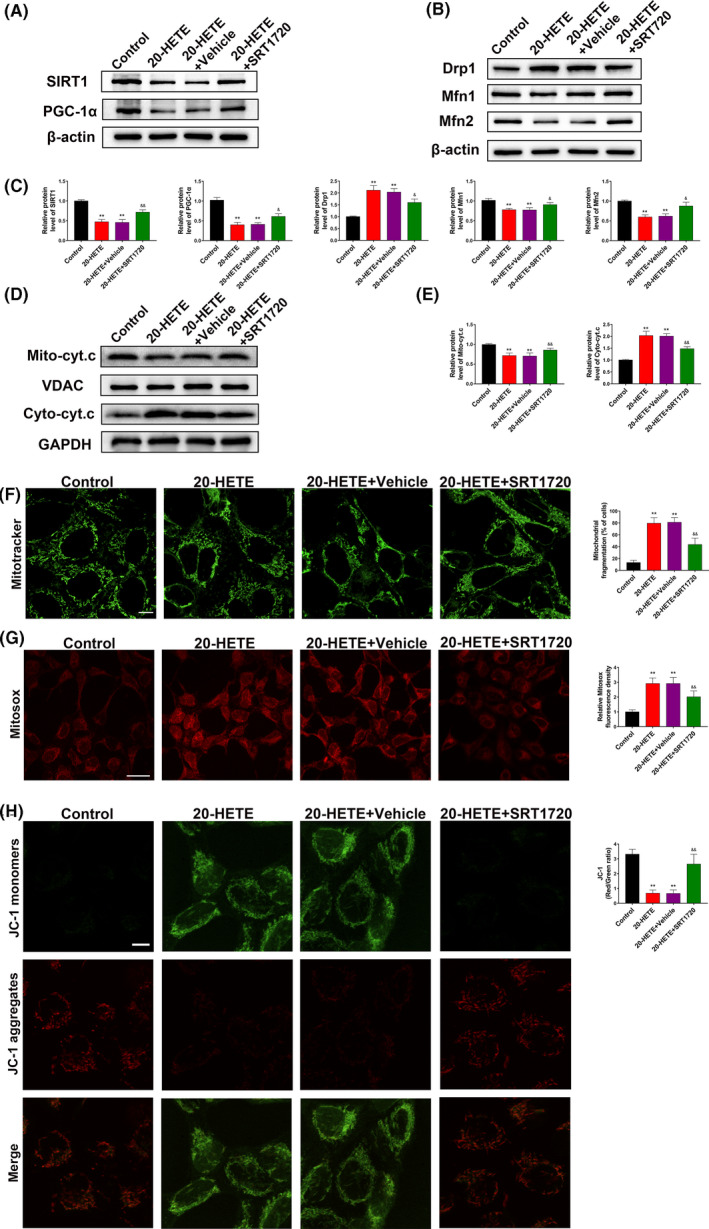
20‐HETE alters normal mitochondrial morphology and function via the SIRT1/PGC‐1α pathway in vitro. A, C, Western blots and statistical analysis of the levels of SIRT1 and PGC‐1α in each group. B, C, Western blots and statistical analysis of the levels of Drp1, Mfn1 and Mfn2 in each group. D, E Western blots and statistical analysis of the levels of mitochondrial and cytoplasmic cytochrome *c* in each group. F, Representative MitoTracker fluorescence images illustrating mitochondrial morphology. Scale bar: 10 μm. G, Representative MitoSOX fluorescence images of mitochondria‐derived ROS. Scale bar: 50 μm. H, Representative fluorescence staining of JC‐1 aggregates (red)/JC‐1 monomers (green) illustrating the MMP. Scale bar: 10 μm. n = 6 for each group. **P < .01 vs control group, ^&^ *P* < .05 and ^&&^ *P* < .01 vs 20‐HETE + vehicle group. Values are presented as the mean ± SD

To validate the involvement of the SIRT1/PGC‐1α pathway in 20‐HETE–induced mitochondrial dysfunction, the alterations in mitochondrial morphology and function in primary neurons after treatment with 20‐HETE were evaluated in vitro. Based on the levels of 20‐HETE detected in the perilesional cortex after TBI (Figure 2B), primary neurons were treated with 10 μM 20‐HETE for 24 hours in vitro, while the 20‐HETE + SRT1720 group was pretreated with SRT1720 (5 μM) for 2 hours. After treatment with 20‐HETE, SIRT1 and PGC‐1α were significantly downregulated. SRT1720 (5 μM) reversed the SIRT1 and PGC‐1α downregulation induced by 20‐HETE (Figure 7A,C). SRT1720 also partially reversed the increase in the level of Drp1 and decrease in the levels of Mfn1 and Mfn2 after 20‐HETE treatment (Figure 7B,C) and inhibited the increase in the level of cytosolic cytochrome *c* released from the mitochondria (Figure 7D,E). MitoTracker staining showed that mitochondrial fragmentation increased after treatment with 20‐HETE and that these effects were reduced by treatment with SRT1720 (Figure 7F). MitoSOX Deep Red staining was used to evaluate mitochondrial ROS levels, and JC‐1 staining was performed to assess the MMP. 20‐HETE significantly increased ROS production and decreased the MMP; these effects were mitigated by treatment with SRT1720 (Figure 7G,H). These results indicate that 20‐HETE induces changes in the normal morphology and function of mitochondria via regulation of the SIRT1/PGC‐1α pathway.

### 3.8 Plasma 20‐HETE levels in patients with TBI correlate with long‐term outcome

**FIGURE 8:**
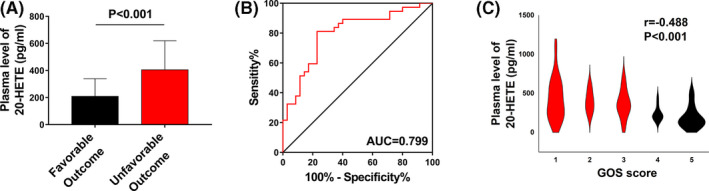
Plasma 20‐HETE levels in patients with TBI correlate with long‐term outcome. A, Plasma 20‐HETE levels are increased in patients with poor outcome compared to those in patients with good outcome (*P* < .001). B, ROC analysis of the discriminative ability of plasma 20‐HETE levels for risk of poor outcome in patients with TBI. Area under the curve: 0.799; optimal cut‐off point: 254.26 pg/mL; sensitivity: 81.1%; specificity: 77.1%. C, Linear correlation between plasma 20‐HETE levels and short‐term neurological outcome assessed using GOS scores of patients with TBI. *r* = −.488, *P* < .001. Values are presented as the mean ± SD

To confirm the translational relevance of our data, we analysed plasma 20‐HETE levels in patients with TBI. A total of 72 patients were included in our study. Plasma 20‐HETE levels were significantly higher in patients with unfavourable outcomes than in those with favourable outcomes (*P* < .001; Figure 8A). Receiver operating curve (ROC) analysis was used to assess the discriminative ability of plasma 20‐HETE levels for outcome. The cut‐off value of 20‐HETE corresponding to the best discrimination of unfavourable outcome was 254.26 pg/mL (81.1% sensitivity and 77.1% specificity; Figure 8B). The Spearman correlation coefficient was calculated to determine the correlation between plasma 20‐HETE level and outcome identified by 6‐month GOS score. The results indicated a negative correlation between plasma 20‐HETE levels and 6‐month GOS score (*r* = −.488 and *P* < .001; Figure 8C). The baseline clinical characteristics of the entire cohort are shown in Table 1. According to the results of univariate and logistic analyses, elevated plasma 20‐HETE level (OR = 1.012, 95% CI: 1.005‐1.019, adjusted *P* = .001; Table 1) as a continuous variable was an independent predictor of unfavourable outcome. These clinical results revealed that plasma 20‐HETE levels were closely associated with outcome in patients with TBI.

**TABLE 1:** Characteristics of the study population

	Study population (n = 72)	Favourable outcome (n = 35)	Unfavourable outcome (n = 37)	Crude OR (95% CI )	Crude *P*‐value	Adjusted OR (95% CI)	Adjusted *P*‐value
Demographic							
Age (y), mean (SEM)	54.53 (1.57)	51.26 (2.29)	57.62 (2.05)	1.039 (1.001, 1.079)	**.047**		
Male, n (%)	50 (69.44)	26 (74.29)	24 (64.86)		.387		
GCS, mean (SEM)	9.26 (0.46)	11.6 (0.57)	7.05 (0.49)	0.668 (0.555, 0.804)	**<.001**	0.665 (0.515, 0.859)	**.002**
Abnormal pupil reaction, n (%)	40 (55.56)	13 (37.14)	27 (72.97)	4.569 (1.684, 12.399)	**.003**		
Mechanism of injury							
Motor vehicle, n (%)	29 (40.28)	14 (40.00)	15 (40.54)		.958		
Fall, n (%)	38 (52.78)	19 (54.28)	19 (51.35)				
Strike, n (%)	3 (4.17)	1 (2.86)	2 (5.41)				
Others, n (%)	2 (2.77)	1 (2.86)	1 (2.70)				
Marshall CT grade, mean (SEM)	3.84 (0.17)	3.14 (0.21)	4.56 (0.22)	2.353 (1.152, 3.662)	**<.001**	2.965 (1.438, 6.116)	**.003**
Tracheotomy, n (%)	19 (26.39)	4 (11.43)	15 (40.54)	5.284 (1.543, 18.093)	**<.001**		
Laboratory biochemical examinations							
Abnormal RBC, n (%)	20 (27.78)	9 (25.71)	11 (29.73)		.704		
Hyperglycaemia, n (%)	17 (23.61)	4 (11.43)	13 (35.14)	4.198 (1.214, 14.519)	**.023**		
Abnormal AST, n (%)	37 (51.39)	10 (28.57)	27 (72.97)	6.750 (2.406, 18.938)	**<.001**		
Abnormal ALT, n (%)	21 (29.17)	6 (17.14)	15 (40.54)	3.295 (1.100, 9.870)	**.033**		
Coagulopathy, n (%)	15 (20.83)	5 (14.29)	10 (27.03)		.189		
20‐HETE (pg/mL), mean (SEM)	309.92 (23.90)	209.17 (21.91)	405.22 (35.27)	1.007 (1.003, 1.011)	**<.001**	1.012 (1.005, 1.019)	**.001**

*P* < 0.05 are significance of bold values

## 4 DISCUSSION

Traumatic brain injury is a serious condition with a considerable global burden, and while the incidence of TBI is increasing, treatment options are limited.^33^ In this study, non‐targeted metabolomic analysis of TBI‐affected and normal brain tissue was used to identify novel therapeutic targets. Considering the important role of neuronal apoptosis in the prognosis of TBI, the detrimental effect of 20‐HETE was investigated in neurons after TBI using samples from patients, an animal model and primary cells.

**FIGURE 9:**
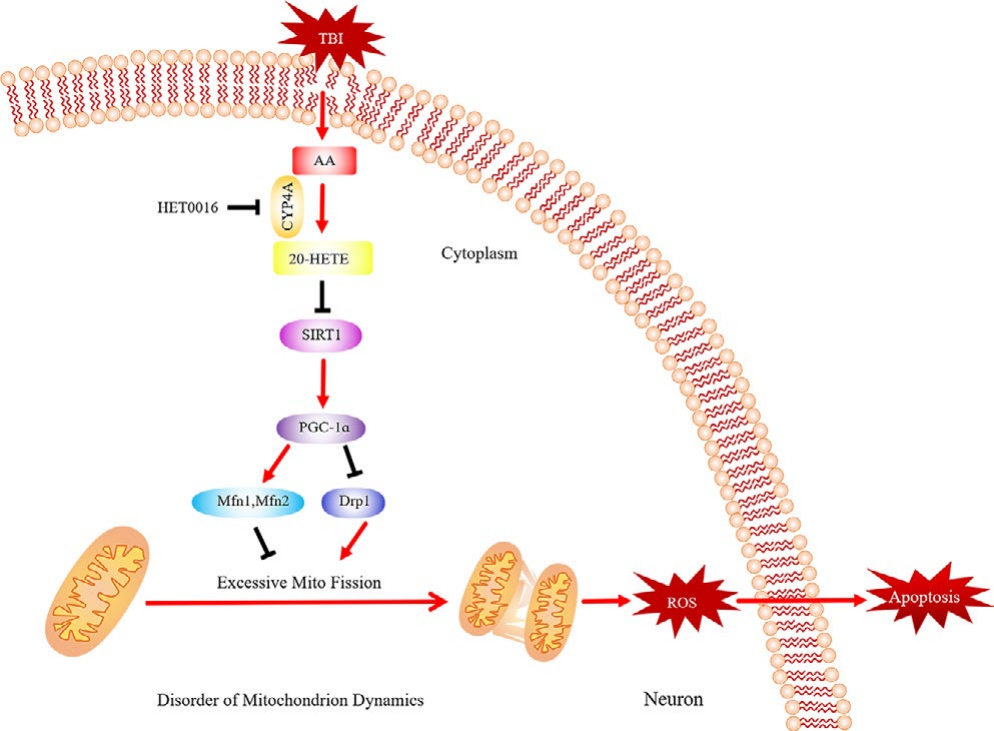
The proposed mechanism of 20‐HETE–induced alterations of mitochondrial dynamics in neurons. 20‐HETE is synthesized from AA by CYP4A, which is inhibited by HET0016. The altered mitochondrial dynamics induced by 20‐HETE activate oxidative stress and subsequently result in neuronal apoptosis mediated by inhibition of the SIRT1/PGC‐1α signalling pathway

Our results demonstrate that 20‐HETE induces apoptosis in mouse cortical neurons after TBI and that 20‐HETE may be a novel target for therapeutic intervention in TBI. The mechanism of 20‐HETE–induced altered mitochondrial dynamics in neurons was investigated (Figure 9), and our experimental data confirmed our hypotheses: (a) the non‐targeted metabolic profiling results indicated that 20‐HETE levels increased significantly in the perilesional cortex after TBI in mice and CYP4A was present exclusively in neurons; (b) HET0016 treatment improved neurological functional deficits, alleviated neural apoptosis and oxidative stress, and protected neuronal ultrastructure after TBI; (c) 20‐HETE disrupted mitochondrial morphology and functioning via regulation of the SIRT1/PGC‐1α signalling pathway in vivo and in vitro; and (d) the plasma level of 20‐HETE in patients with TBI were correlated with long‐term outcome.

The results of non‐targeted metabolic profiling indicated that the level of 20‐HETE was significantly increased after TBI. 20‐HETE affects neuronal survival in ischaemic and haemorrhagic stroke^15^, ^32^, ^34^ and thus may play a detrimental role in TBI as well. A large body of evidence indicates that CYP4A is widely expressed in neurons and can be upregulated by oxidative stress.^35^, ^36^ We found that CYP4A was present mainly in neurons and that the 20‐HETE concentration was significantly increased in the early stage of TBI (within 24 hours), suggesting that excessive levels of 20‐HETE may be a cause of neuronal damage and apoptosis.

The mechanism by which an increase in 20‐HETE levels induced damage was determined by administering HET0016 in mice after TBI. Treatment with HET0016 reduced oxidative stress and neural death in vivo in the early post‐TBI period. This HET0016‐induced reduction in neuronal death may be related to a decrease in oxidative stress. Additional studies are needed to determine whether HET0016 has a protective effect in late post‐TBI stages as well. To determine the mechanism of HET0016‐dependent neuroprotection after TBI, transmission electron microscopy was used to analyse the ultrastructural changes in neurons. 20‐HETE induces apoptosis in cardiac myocytes in a mitochondrial superoxide‐dependent manner.^37^, ^38^ In the present study, in addition to mitochondrial swelling, loss of mitochondrial cristae and membrane integrity were detected in neurons after TBI; these effects were partially reversed by HET0016 treatment, indicating that 20‐HETE–induced neural death and oxidative stress may be attributed to mitochondrial dysfunction.

Mitochondria are highly dynamic organelles that are involved in the regulation of energy production, ROS generation and apoptosis,^39^ and continuously undergo fission and fusion.^40^ Drp1 is one of the key mediators of mitochondrial fission, and Mfn1 and Mfn2 are two mitochondrial fusion regulators; all three proteins are regulated by PGC‐1α.^41^, ^42^, ^43^ In our study, HET0016 treatment reversed the increase in the expression of Drp1 and decrease in the expression of Mfn1 and Mfn2 after TBI in vivo, indicating that HET0016 can correct the balance of mitochondrial fission and fusion. Seven SIRT family members have been determined in mammals. SIRT1 and SIRT2 are localized in the nucleus and cytoplasm. SIRT3, SIRT4 and SIRT5 are mitochondrial, while SIRT6 and SIRT7 are nuclear.^44^ SIRT1 is the most important member of the sirtuin family and is associated with the regulation of mitochondrial biogenesis, inflammation and cell senescence. Although primarily a nuclear protein, extensive research has proved that SIRT1 can activate PGC‐1α, which contributes to enhanced mitochondrial biogenesis.^45^, ^46^ Angiotensin II–induced cardiomyocyte apoptosis is closely associated with mitochondrial fission, which in turn is closely associated with the SIRT1 signalling pathway.^47^ Angiotensin II increased CYP4A expression and 20‐HETE production; furthermore, treatment of cardiomyocytes with HET0016 significantly alleviates angiotensin II–induced ROS production, mitochondrial dysfunction and apoptosis, indicating that 20‐HETE may play a key role in angiotensin II–induced cardiomyocyte apoptosis.^38^ Thus, we hypothesized that 20‐HETE might contribute to apoptosis in a mitochondria‐dependent manner by regulating the SIRT1/PGC‐1α signalling pathway. Our data demonstrate that HET0016 treatment reversed the decrease in SIRT1 and PGC‐1α levels after TBI in vivo. Moreover, 20‐HETE inhibited SIRT1/PGC‐1α expression and altered normal mitochondrial function in primary neurons in vitro. The increase in the level of Drp1 and the decrease in the levels of Mfn1 and Mfn2 in the 20‐HETE–treated groups were rectified by SRT1720. Additionally, we determined whether other SIRT family members were involved in the process. We found that SIRT2, SIRT3, SIRT4, SIRT5 and SIRT6 to be significantly reduced after TBI, while they could not be reversed by HET0016 treatment, and SIRT7 did not change after TBI or HET0016 treatment (Figure S2A,B). Our results indicated that 20‐HETE produced after TBI may mainly regulate mitochondrial function and cell proliferation via the SIRT1/PGC‐1α pathway.

To substantiate the translational relevance of our preclinical data, plasma 20‐HETE levels were assayed in patients with TBI. Our study is the first to assay plasma 20‐HETE levels in these patients, and the results indicated that plasma 20‐HETE levels were significantly higher in patients with unfavourable outcomes than in those with favourable outcomes. Furthermore, plasma 20‐HETE levels and 6‐month GOS scores were found to be negatively correlated. These data are consistent with the results of the in vivo and in vitro experiments, indicating that 20‐HETE is a negative regulator of TBI outcomes. Additional studies are needed to determine whether 20‐HETE is associated with neurological deterioration, such as an increase in intracranial pressure and progressive intracranial haemorrhage. Our data indicate that 20‐HETE significantly inhibits the expression of SIRT1; thus, plasma SIRT1 levels were also assayed in patients with TBI. Our results showed that SIRT1 levels were higher in patients with favourable outcomes than in those with unfavourable outcomes (1.145 ng/mL vs 0.883 ng/mL, *P* = .02; Figure S3), suggesting that SIRT1 is a positive regulator of TBI outcome.

Additionally, considering the role of 20‐HETE as an angiogenesis mediator,^48^ we determined whether 20‐HETE inhibition delays angiogenesis after TBI. Studies have demonstrated that HET0016 treatment reduces neurological deficits and inflammatory responses without inhibiting angiogenesis in intracerebral haemorrhage.^15^, ^49^ Our data showed that HET0016 did not inhibit angiogenesis in vivo at day 28 after TBI. Expression levels of VEGF were decreased 7, 14 and 28 days after TBI compared with that in the sham group. HET0016 treatment did not significantly alter the level of VEGF expression compared with that in vehicle‐treated mice (Figure S4). In our study, we proved that high levels of 20‐HETE were produced by neurons and may cause neuronal apoptosis after TBI. However, it remains to be clarified whether 20‐HETE affects astrocytes, microglia and other cells in the microenvironment of TBI and contributes to secondary injury.

In conclusion, the results of this translational study indicate that 20‐HETE plays a detrimental role in TBI by inducing neuronal apoptosis. At the molecular level, 20‐HETE may modulate mitochondrial dysfunction in neurons via the SIRT1/PGC‐1α signalling pathway. The data from our study set the ground for subsequent studies to validate the inhibition of 20‐HETE as a novel strategy for the treatment of TBI.

## CONFLICTS OF INTEREST

The authors declare no conflicts of interest.

## AUTHOR CONTRIBUTIONS

Yan Qu, Shunnan Ge and Dayun Feng developed hypotheses and designed experiments. Wenxing Cui and Xun Wu performed experiments and wrote the manuscript. Yingwu Shi and Shunnan Ge contributed to the collection and analysis of patient samples. Wei Guo and Jianing Luo helped to draft the manuscript. Haixiao Liu, Yong Du and Longlong Zheng analysed data. Ping Wang and Qiang Wang collected the clinical data.

## ETHICAL APPROVAL

This study was approved by the Ethics Committee of Tangdu Hospital, Fourth Military Medical University.

## Supporting information

Fig S1

Fig S2

Fig S3

Fig S4

## Data Availability

The data used and analysed in this study are available from the corresponding author on reasonable request.
