# Supplementary figures and images for: 20‐HETE synthesis inhibition attenuates traumatic brain injury–induced mitochondrial dysfunction and neuronal apoptosis via the SIRT1/PGC‐1α pathway: A translational study
Source: Cell Prolif. 2020 Dec 13;54(2):e12964. doi: 10.1111/cpr.12964 (PMC7848954; doi:10.1111/cpr.12964)

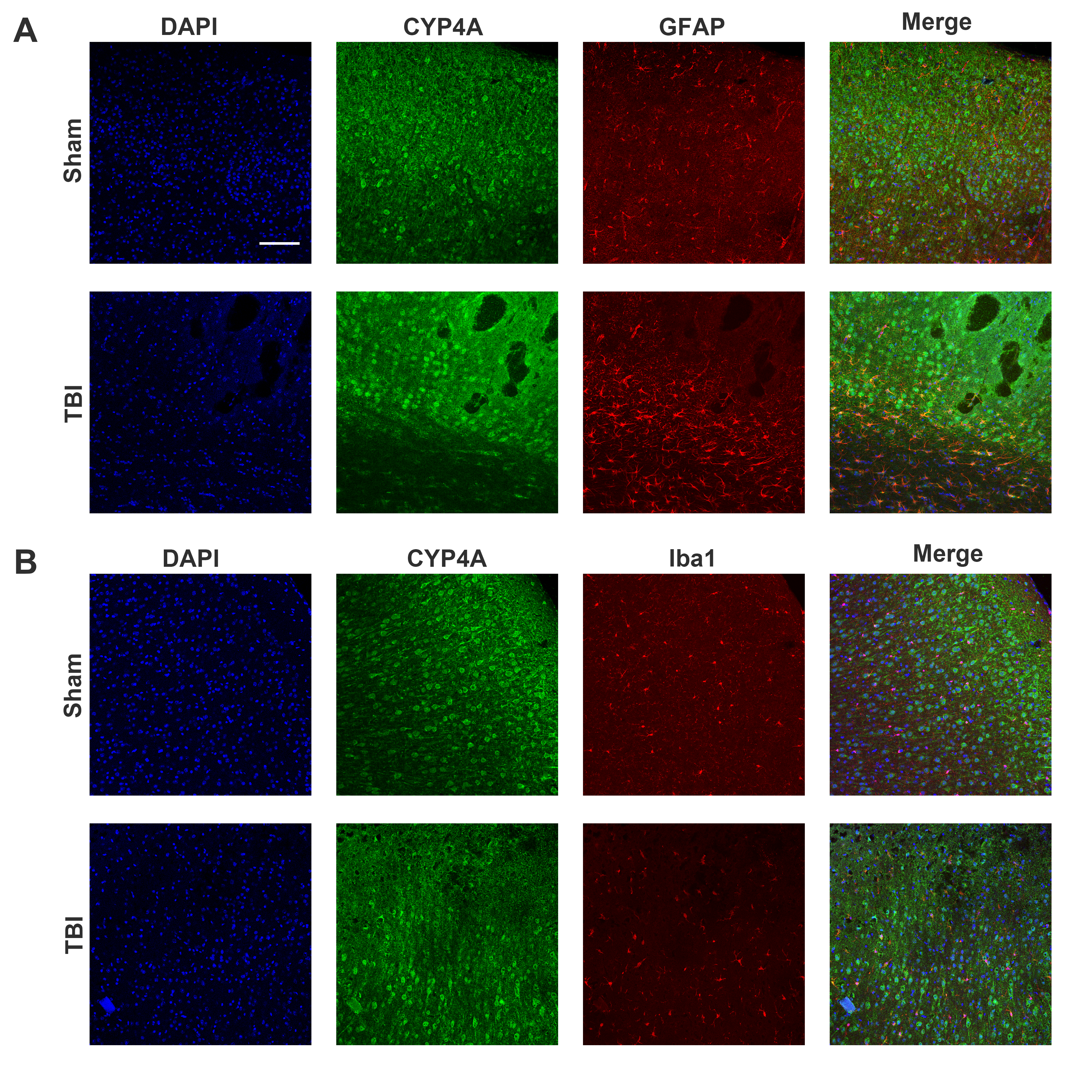

Supplement: Supplementary file 1 — Fig S1 [file CPR-54-e12964-s001.tif]

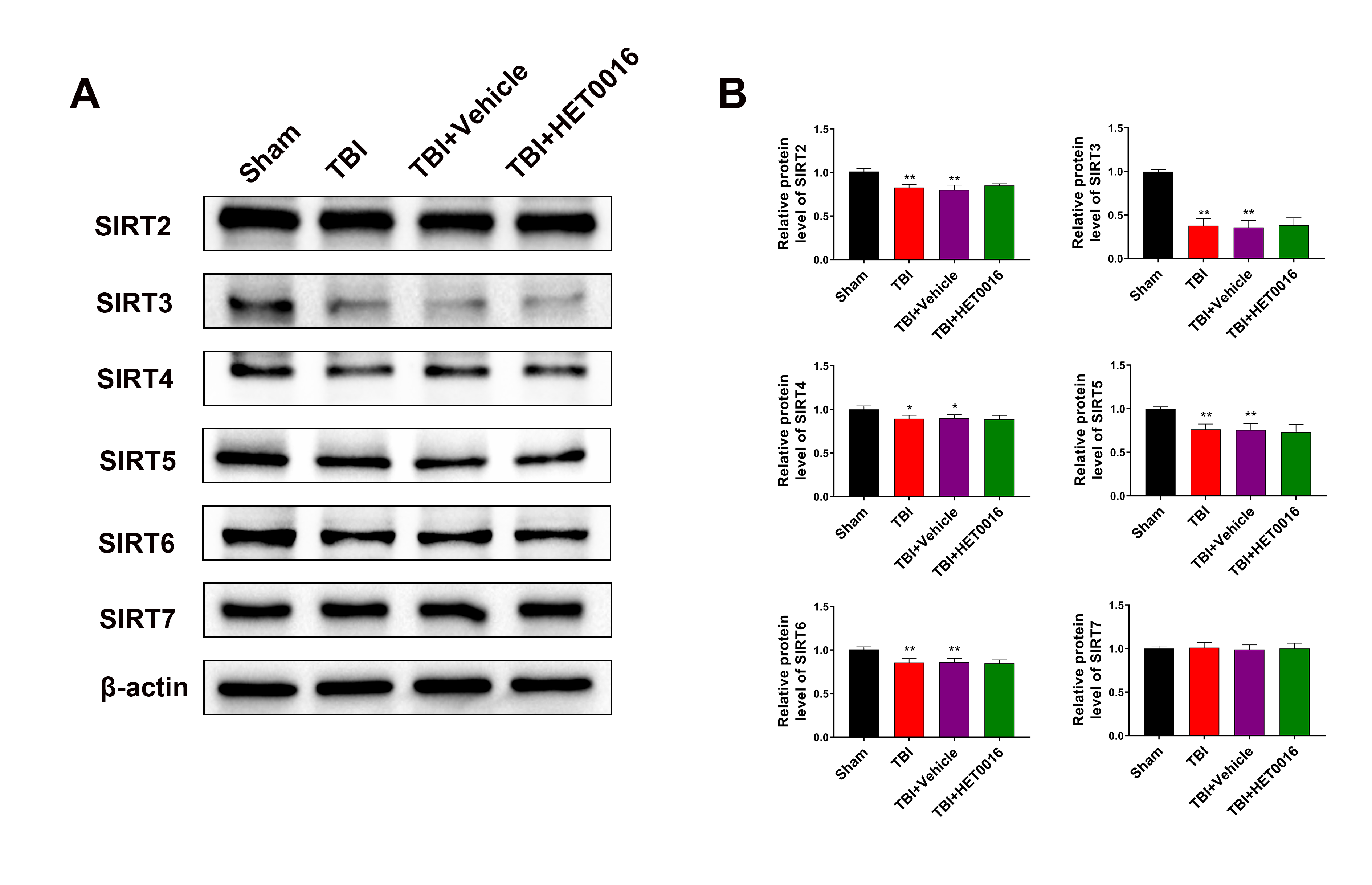

Supplement: Supplementary file 2 — Fig S2 [file CPR-54-e12964-s002.tif]

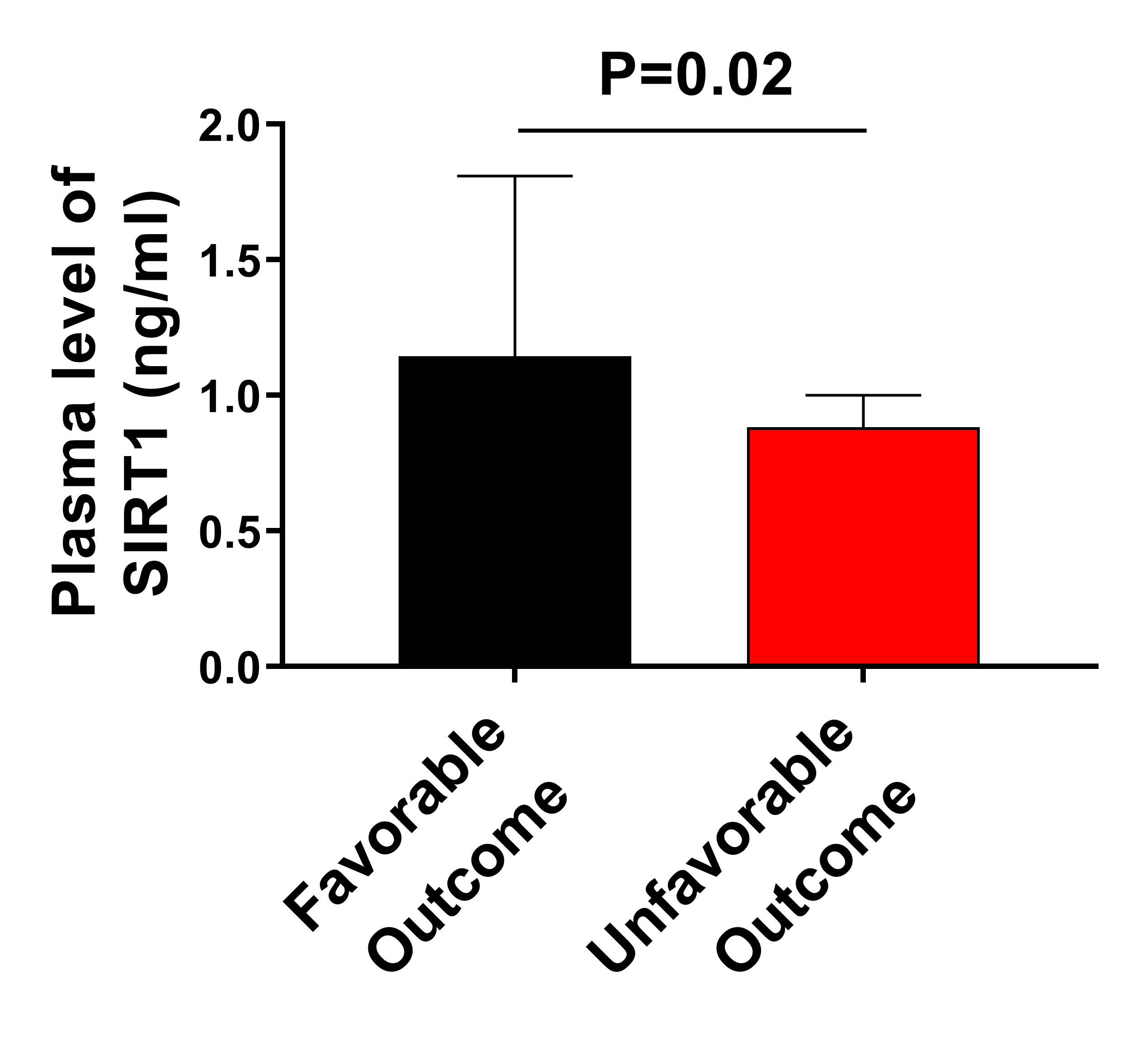

Supplement: Supplementary file 3 — Fig S3 [file CPR-54-e12964-s003.tif]

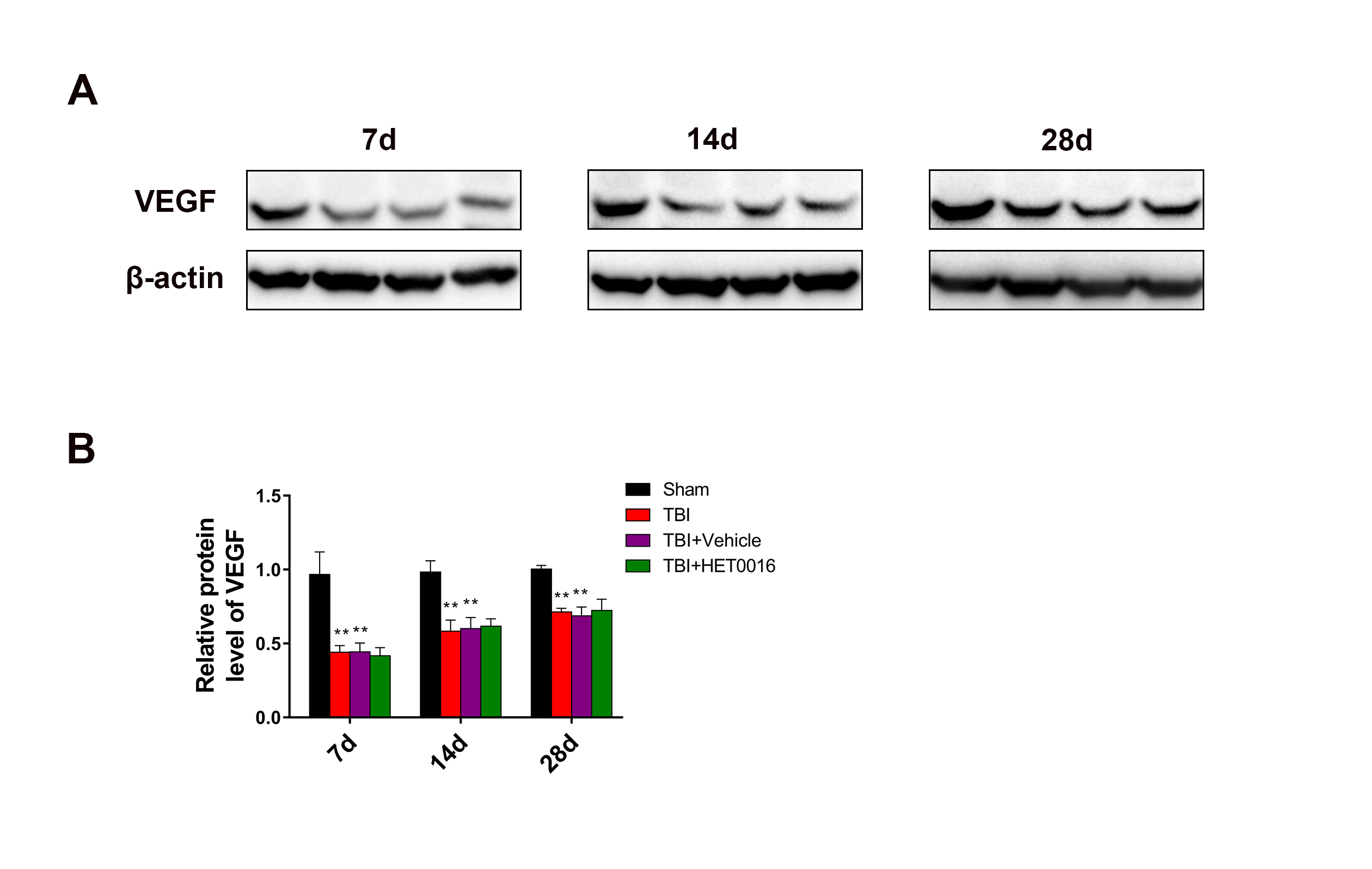

Supplement: Supplementary file 4 — Fig S4 [file CPR-54-e12964-s004.tif]
